# Extraction of short chain chitooligosaccharides from fungal biomass and their use as promoters of arbuscular mycorrhizal symbiosis

**DOI:** 10.1038/s41598-021-83299-6

**Published:** 2021-02-15

**Authors:** Andrea Crosino, Elisa Moscato, Marco Blangetti, Gennaro Carotenuto, Federica Spina, Simone Bordignon, Virginie Puech-Pagès, Laura Anfossi, Veronica Volpe, Cristina Prandi, Roberto Gobetto, Giovanna Cristina Varese, Andrea Genre

**Affiliations:** 1 Department of Life Science and Systems Biology, University of Turin, 10125 Turin, Italy; 2 Department of Chemistry, University of Turin, 10125 Turin, Italy; 3 Laboratoire de Recherche en Sciences Végétales, Université de Toulouse, CNRS, UPS, 31320 Castanet-Tolosan, France

**Keywords:** Arbuscular mycorrhiza, Polysaccharides, Fungal host response, Biochemical assays, Liquid chromatography, Mass spectrometry, Fluorescence imaging, Microbiology techniques, Ca2+ imaging, Confocal microscopy

## Abstract

Short chain chitooligosaccharides (COs) are chitin derivative molecules involved in plant-fungus signaling during arbuscular mycorrhizal (AM) interactions. In host plants, COs activate a symbiotic signalling pathway that regulates AM-related gene expression. Furthermore, exogenous CO application was shown to promote AM establishment, with a major interest for agricultural applications of AM fungi as biofertilizers. Currently, the main source of commercial COs is from the shrimp processing industry, but purification costs and environmental concerns limit the convenience of this approach. In an attempt to find a low cost and low impact alternative, this work aimed to isolate, characterize and test the bioactivity of COs from selected strains of phylogenetically distant filamentous fungi: *Pleurotus ostreatus*, *Cunninghamella bertholletiae* and *Trichoderma viride*. Our optimized protocol successfully isolated short chain COs from lyophilized fungal biomass. Fungal COs were more acetylated and displayed a higher biological activity compared to shrimp-derived COs, a feature that—alongside low production costs—opens promising perspectives for the large scale use of COs in agriculture.

## Introduction

Agriculture is experiencing an urgent need to shift toward low-input practices that can be integrated with the environment and aim at food safety for the growing human population. Arbuscular mycorrhizal (AM) fungi can play a key role in this scenario^4^. AM is the most widespread plant symbiosis, in terms of geographical distribution and phylogenetic coverage in the plant kingdom^1^. In this mutualistic symbiosis, part of the plant sugars and lipids are fed to the fungus^2^, while soil mineral nutrients and water are scavenged by the extraradical mycelium and transferred to the plant, improving its fitness^1^.

AM symbiosis plays a central ecological role in the functioning of natural ecosystems, but most crop plants establish this beneficial interaction too^4^. In this context, the potential use of AM fungi in sustainable production under low chemical input conditions is currently raising a broad interest in agronomic context^3^. Promoting and maintaining functional and persistent mycorrhizal symbioses in crop fields to improve soil quality and productivity requires proper management of agroecosystems with strategies that include shallow tillage, low fertilizer input, perennial crop development, as well as AM promotion with fungal inoculation and treatments that stimulate symbiosis establishment^4,5^.

Plants interact with a multitude of microbes and their ability to recognize them and deploy appropriate responses is largely based on the recognition of microbe-specific molecules known as microbe-associated molecular patterns^6^. Among them, long-chain oligomers of chitin—which is the main fibrillar component of fungal cell wall—are strong elicitors of plant defense responses^7–9^. The perception of long-chain chito-oligomers such as chitooctaose (composed of 8 N-acetyl-glucosamine residues) activates powerful defense strategies of the plant, such as the release of chitinases, phytoalexins, reactive oxygen species and callose deposition in the cell wall^10^.

Other chitin-related molecules are known to play a signalling role in symbiosis as so-called mycorrhizal factors, or Myc-factors. Among them, tetra/penta-chito-oligosaccharides (CO4-5) activate symbiotic signalling in all tested dicot and monocot AM hosts^11,12^. The plant signal transduction pathway mediating Myc-factor recognition includes the activation of intense oscillations in Ca^2+^ concentration in the nuclei of root epidermal cells^13^, which is now commonly used as a hallmark of Myc-factor perception^13,14^.

Myc-factors activate plant symbiotic responses ranging from gene regulation to metabolic and developmental changes^15,16^ that have been defined as part of an anticipation program preparing the host to a successful association^17^. Furthermore, the application of exogenous COs was recently demonstrated to stimulate lateral root development and branching in *Oryza sativa*^13^ and AM establishment in the model legume *Medicago truncatula*^4^, paving the way to possible use of CO treatments to promote AM in agricultural applications.

Currently, commercial COs of different length are obtained from fishing waste processing industries, mainly through hydrolysis of shrimp shell-derived chitin and chitosan^18,19^. Seasonal availability, environmental concerns and purification costs are the main drawbacks of the use of shrimp-derived COs in large-scale applications. An alternative, more sustainable and cheaper source of COs are fungal biomass wastes from fermentation industries. In fact, in spite of having lower chitin content than crustaceans (10–26% as a chitin-glucan complex), fungal biomass does not need aggressive acid treatments—normally required for the removal of calcium carbonate and other minerals from crustacean shells—prior to CO extraction. Furthermore fungal biomass production is not subject to seasonal and regional limitations^19^.

Here we present a protocol (see Supplementary Fig. S1 online) to efficiently extract COs from fungal biomass of different origin (*Pleurotus ostreatus*, *Cunninghamella bertholletiae* and *Trichoderma viride)*; we confirm the chemical properties of the extracted COs and demonstrate their stronger biological activity as promoters of AM symbiosis when compared with commercial COs.

## Results

### Extraction yields

When grown on a standard medium (liquid malt extract), *P. ostreatus* provided the highest amount of mycelium with a yield of 10 g/L. The quantity of products obtained after the extraction of chitosan and chitin and after chitin acid hydrolysis are shown in Table 1. The amount of chitin extracted varied among species, with *P. ostreatus* biomass displaying the highest yield in chitin (24.7%). All tested fungi presented a low yield in chitosan, ranging between 0.26 and 0.04% of the starting biomass.

**Table 1:** Chitin, chitosan and CO yield from each of the three fungi.

	Chitin		Chitosan		COs	
	g	%	g	%	g	%
*Pleurotus ostreatus*	3.7	24.7	0.006	0.04	0.202	6.70
*Cunninghamella bertholletiae*	1.2	8.30	0.027	0.18	0.362	29.20
*Trichoderma viride*	2.6	17.30	0.039	0.26	0.042	1.70

Yields are expressed as both dry mass (g) and percentage values (%) of the starting fungal dry biomass (for chitin and chitosan) or the starting chitin amount (for COs).

Due to the very limited amount of extracted chitosan, only chitin was treated by acid hydrolysis for CO production. The yield in putative COs was 6.70% of the starting chitin amount for *P. ostreatus*, 29.20% for *C. bertholletiae* and 1.70% for *T. viride*.

Altogether, putative CO yield resulted to be 1.65% of the original mycelium dry weight for *P. ostreatus*, 3.69% for *C. bertholletiae* and 0.30% for *T. viride* (Table 1). By combining growth speed, biomass, chitin and CO yield and lower CO polymerization (max CO6), we decided to use COs from *P. ostreatus*, which is also considered as a GRAS (Generally Recognized As Safe) organism, for all subsequent pot culture analyses.

### Extracted samples contain COs of the expected size and acetylation degree

Direct infusion mass spectrometry (DIMS) indicated that the extracted samples contained acetylated and de-acetylated COs with length comprised between 2 and 7 residues (see Supplementary Fig. S2 online). Pseudo-molecular ions [M + H^+^] of COs composed of 2 to 7 N-acetyl-glucosamine residues were detected, while double-charged ions and longer chains, if existing, were below the limit of detection of the system.

High-performance liquid chromatography-Mass spectometry (HPLC–MS/MS) analysis of the CO mixture (see Supplementary Fig. S3 online) confirmed that our extracted samples contained fully acetylated, mono-deacetylated and di-deacetylated CO molecules composed of 2 to 5 N-acetyl-glucosamine residues.

**Figure 1:**
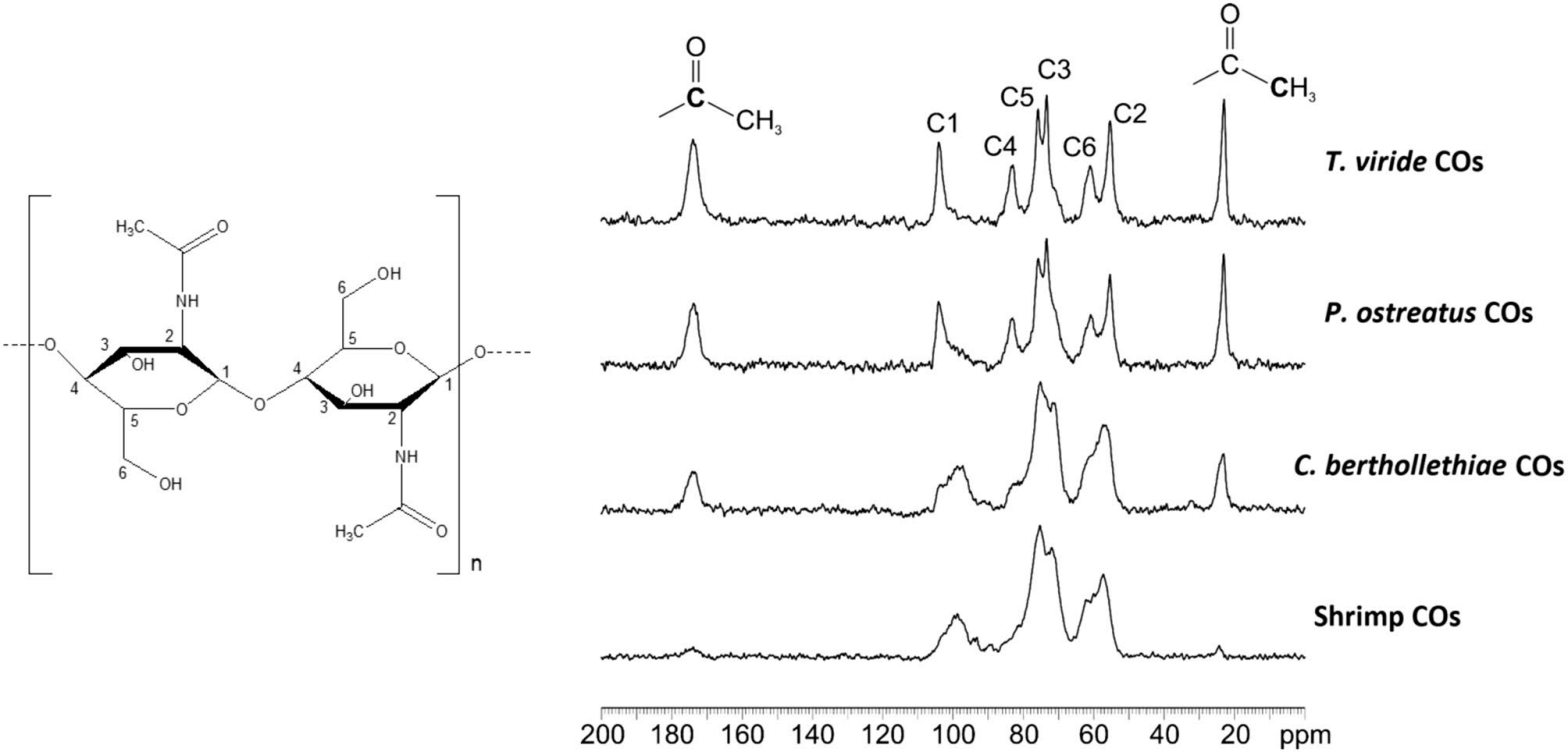
^13^C CPMAS SSNMR spectra of chitooligosaccharides from *T. viride*, *P. ostreatus*, *C. bertholletiae* and shrimps, acquired at room temperature at a spinning speed of 20 kHz. The labels of carbon atoms (C1-C6) refer to the scheme on the left. The spectra indicate that the four samples are characterized by different degrees of acetylation; in particular, this is lowest in shrimp-derived COs and highest in *T. viride-*derived COs.

Solid-State Nuclear Magnetic Resonance (^13^C CPMAS SSNMR) was used to characterize the materials under study, while solution ^1^H NMR was applied for the determination of percent acetylation for each CO sample^20,21^. Figure 1 shows the ^13^C CPMAS SSNMR spectra of COs from *C. bertholletiae*, *P. ostreatus*, *T. viride* and shrimps. Both *C. bertholletiae* and shrimp CO spectra exhibited wider and less sharp spikes than those present in *P. ostreatus* and *T. viride* spectra. These differences indicated that *C. bertholletiae* and shrimp COs were composed of a heterogeneous mix of COs containing a different number of *N*-acetyl-d-glucosamine units, whereas *P. ostreatus* and *T. viride* COs appeared to have a more uniform composition in terms of molecule length. *C. bertholletiae* COs and shrimp shell COs only differed in the acetylation degree, which resulted to be higher in *C. bertholletiae* COs. Overall, SSNMR spectra, albeit intrinsically not quantitative, suggested rather diverse degrees of acetylation for the four samples, with the lowest acetyl peaks for shrimp-derived and the tallest for *T. viride-*derived COs.

**Figure 2:**
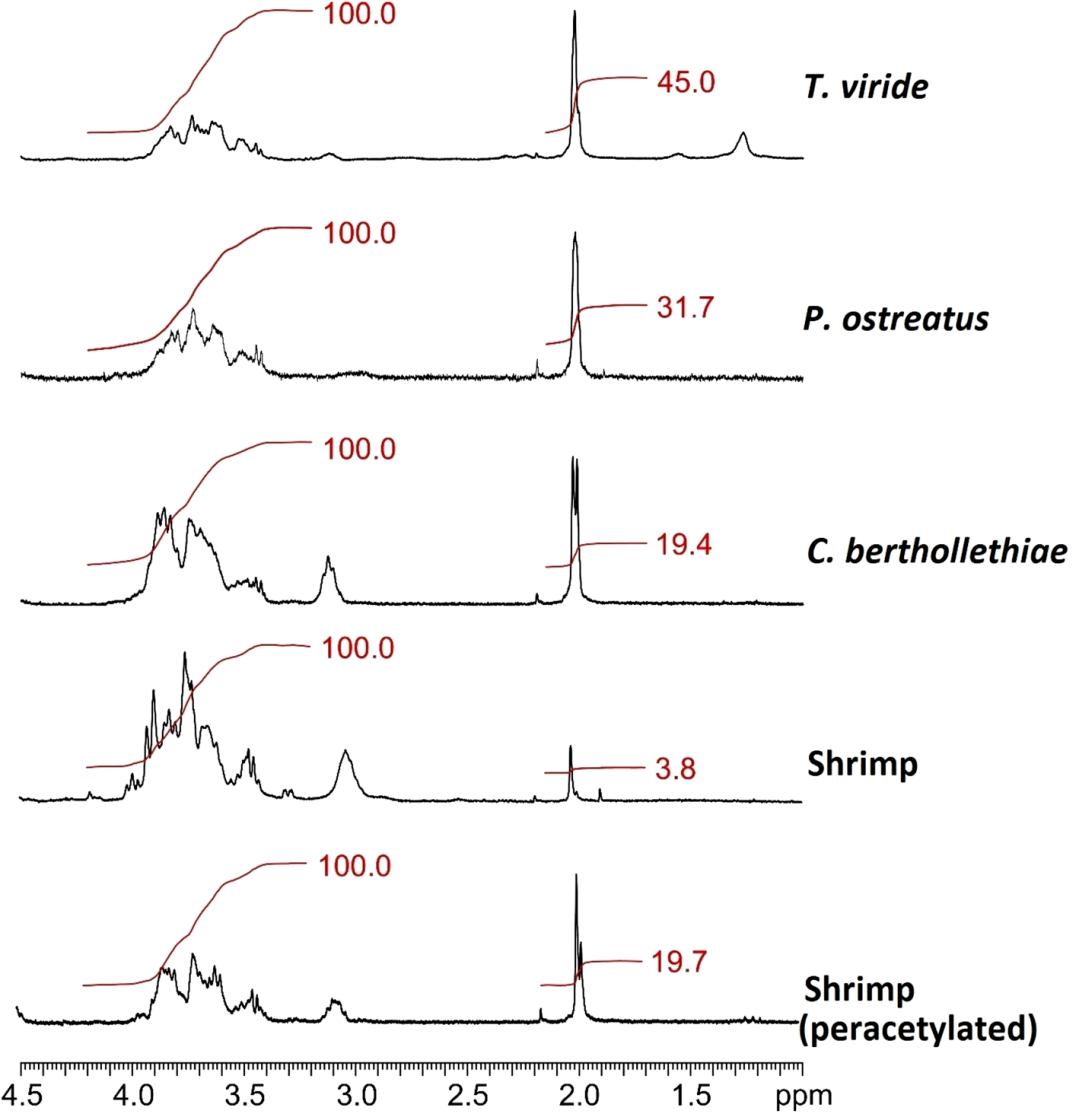
Detail of solution ^1^H NMR spectra shown in Supplementary Fig. S4. Red lines and numbers represent integrals for both chain protons (between 3.2 and 4.2 ppm, normalized at 100.0) and methylic protons of the acetyl groups (at ~ 2.0 ppm).

This prompted us to investigate CO acetylation in more detail through solution ^1^H NMR analyses. To this aim we also prepared a batch of peracetylated shrimp COs by increasing their acetylation degree with a treatment in acetic acid (see “Methods” section). Figure 2 and Supplementary Fig. S4 display the ^1^H spectra of the analyzed samples. In order to quantify the acetylation degree (AD) and compare it among samples, the signals ascribable to protons in the polymeric chain were normalized at 100.0, so that the integrals for acetyl groups could directly represent percent values of acetylation, as reported in Table 2. Solution NMR results confirmed the assumption from the solid-state analysis: *T. viride* resulted to provide the most acetylated COs (AD = 45%), followed by *P. ostreatus* (AD = 31.7%) and *C. bertholletiae* (AD = 19.4%). The analysis also confirmed the efficiency of N-acetylation on shrimp COs, indicating a significant increase in their AD from 3.8 to 19.7%. In conclusion, a combination of chemical analyses confirmed the presence of short chain COs in the extracted samples and the efficiency of our N-acetylation protocol on the otherwise poorly acetylated shrimp-derived commercial COs.

**Table 2:** Percent degree of acetylation of the extracted COs, as resulting from ^1^H NMR spectra.

	*T. viride*	*P. ostreatus*	*C. bertholletiae*	Shrimp	Peracetyl. shrimp
Acetylation (%)	45.0	31.7	19.4	3.8	19.7

### Extracted COs are active as symbiotic signals

**Figure 3:**
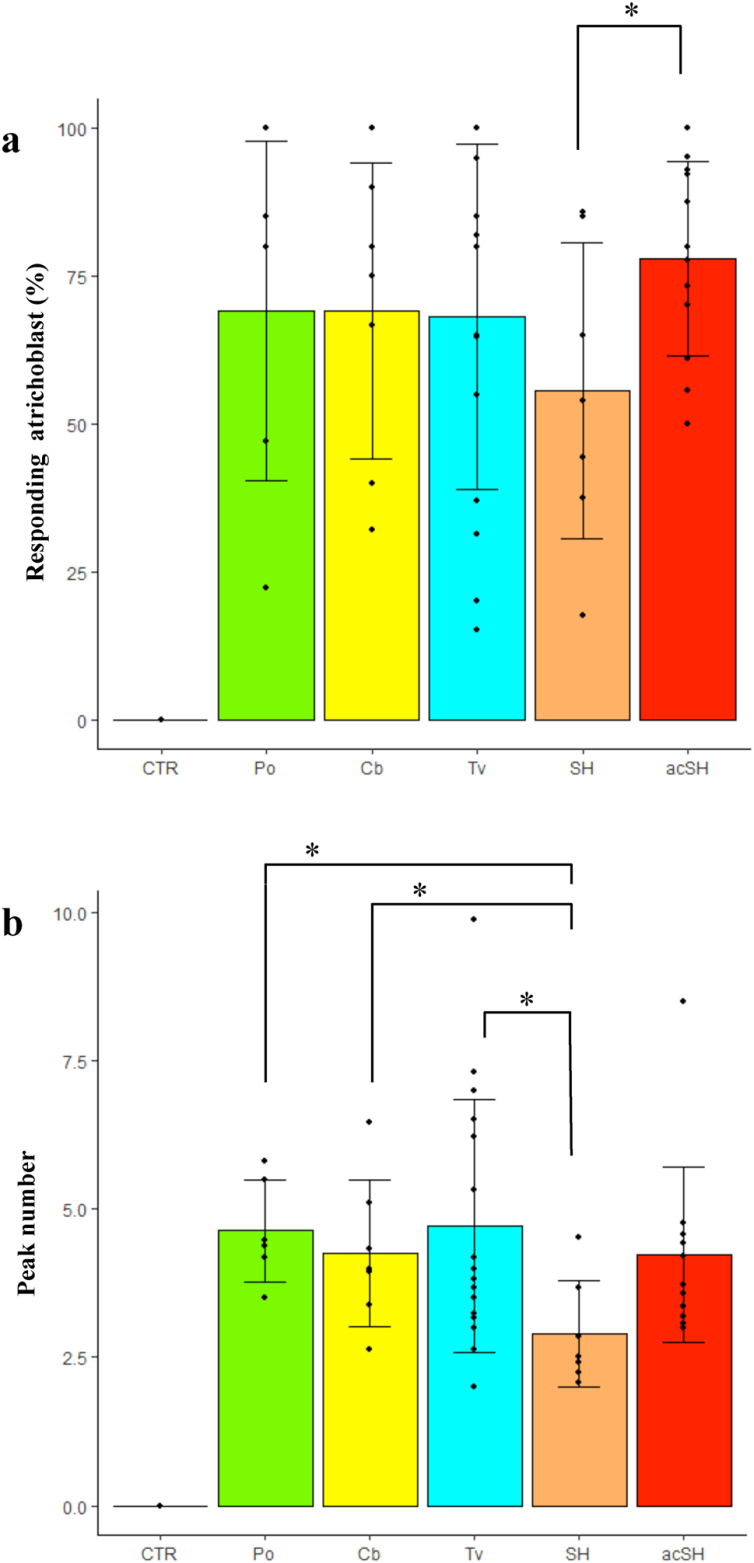
Calcium spiking response to 1 mg/L CO treatment in *M. truncatula* atrichoblasts. All CO solutions activated nuclear calcium spiking, in a comparable number of cells (expressed as percentage in **a**). Nevertheless, the average number of peaks recorded over 30 min (**b**) indicated a significantly stronger response to fungal compared to shrimp COs. Remarkably, this difference was canceled when shrimp COs acetylation degree was increased by peracetylation. CTR = water-treated control; Po = *P. ostreatus* COs; Cb = *C. berthollethiae* COs; Tv = *T. viride* COs; SH = shrimp-derived COs; acSH = peracetylated shrimp-derived COs. A minimum of six biological replicates were evaluated for each treatment. Significant differences are marked by an asterisk (Student’s t test: P < 0.05); the difference between CTR and each CO treatment was highly significant in both analyses (Student's t test: P < 0.005).

Each CO sample was tested for its ability to elicit symbiotic signalling in an AM host plant. To this aim, 1 mg/L CO solutions were applied to *M. truncatula* root organ cultures (ROCs) expressing the NUP-YC2.1 probe (see “Methods” section), and nuclear Ca^2+^ signals were recorded in epidermal cells by live confocal imaging. As expected, control treatments with sterile distilled water did not activate any variation in nuclear Ca^2+^ concentration. On the contrary, repeated nuclear Ca^2+^ oscillations (spiking) were observed in atrichoblasts upon treatment with all CO solutions (Fig. 3).

In order to compare the spiking response elicited by each CO solution, we used the method developed by Russo et al.^22^ for quantifying a few characteristic features of the Ca^2+^ spiking response: the percentage of responding epidermal cells (atrichoblasts) and the average number of peaks per responding cell. This quantitative analysis did not reveal any statistically significant difference in the percent of responding cells between shrimp- and fungal-derived COs. However, fungal COs induced a significantly higher number of peaks, on average, compared to shrimp-derived COs. No significant difference could be observed between fungal samples. Lastly, both the percent of responding atrichoblasts and the average peak number in response to shrimp CO treatment were significantly increased following CO peracetylation.

In short, all tested COs were able to activate symbiotic signalling in *M. truncatula* ROCs, but fungal COs and peracetylated shrimp COs elicited a stronger response than commercial shrimp-derived COs.

### Plant treatment with fungal COs enhances arbuscular mycorrhizal colonization

**Figure 4:**
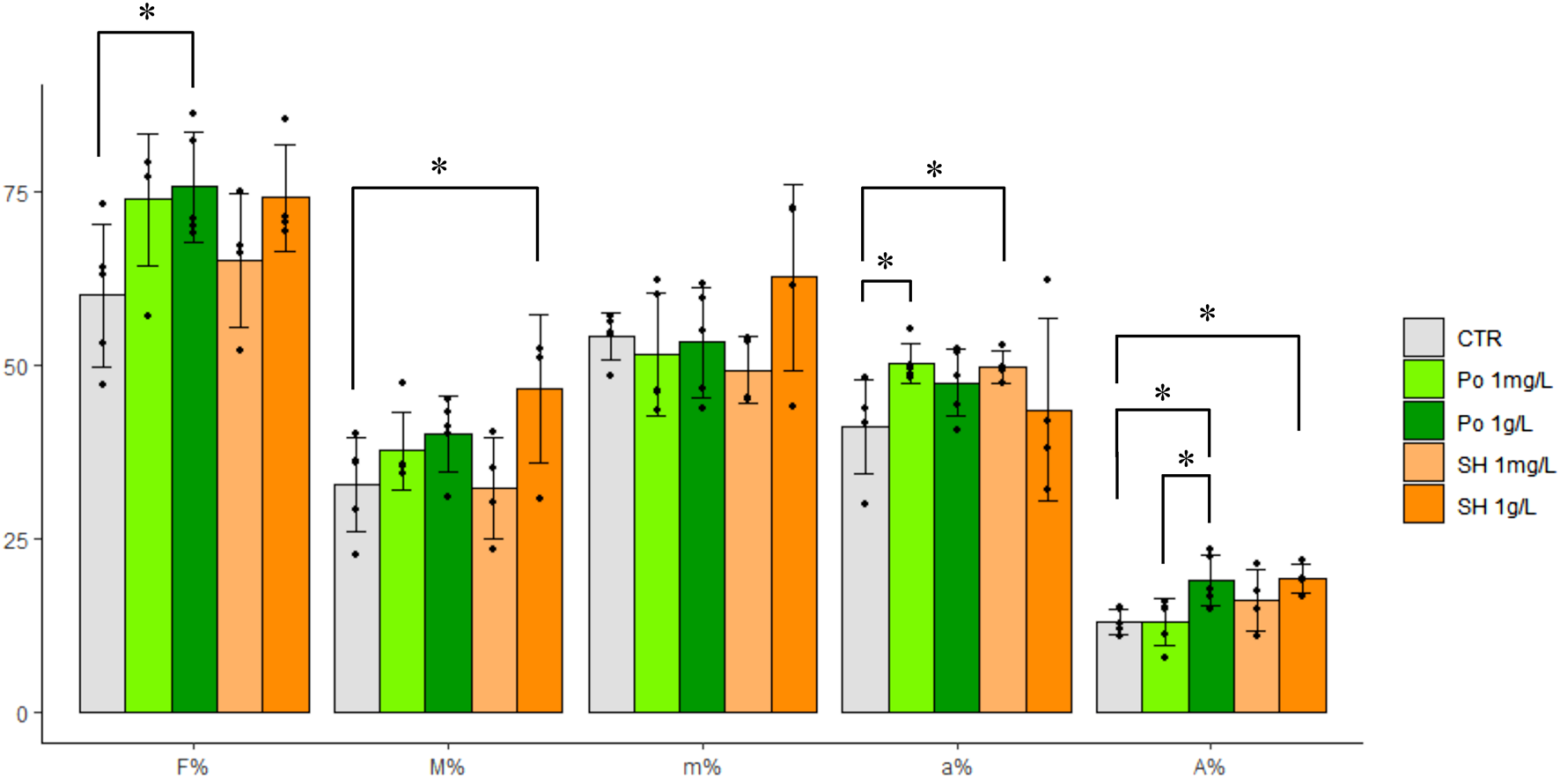
Quantitative analysis of fungal colonization in mycorrhizal *M. truncatula* roots treated with different CO solutions. A general promotion of fungal colonization was induced by all treatments. In more detail, the application of 1 g/L COs from *P. ostreatus* (Po 1 g/L) induced a significant increase in colonization frequency (F%) and arbuscule abundance in the whole root system (A%) compared to water-treated controls. A comparable increase in F% was also obtained using a 1 mg/L solution (Po 1 mg/L), which also produced a significant increase in arbuscule abundance in the colonized areas (a%). By contrast, shrimp-derived COs produced a significant increase in A% and M% (representing the average extension of fungal colonization in the root system), as well as a relevant but statistically non significant elevation of F%, when applied at high concentration (SH 1 g/L), but SH 1 mg/L produced the lowest increase in F% and only a significant increase in arbuscule abundance in the colonized areas (a%). No significant differences were observed in the intensity of the mycorrhization in colonized parts of the root system (m%). In short, *P. ostreatus* COs resulted to be more efficient promoters of AM colonization than shrimp COs, especially when used at low dosage. A minimum of four biological replicates were evaluated for each treatment. Asterisks indicate significant differences (Student’s t test: P < 0.05).

A precise quantification of root colonization by the AM fungus was done according to Trouvelot et al.^23^. As summarized in Fig. 4, the main effect of CO treatment was a general increase in all parameters describing root colonization, in agreement with Volpe et al.^4^. In more detail, a 1 g/L solution of *P. ostreatus* COs produced a significant increase in colonization frequency (F%) and arbuscule abundance in the whole root system (A%). A more diluted treatment (1 mg/L) with *P. ostreatus* COs generated an analogous (albeit not statistically confirmed) trend on F%, and had a major impact on arbuscule abundance within the colonized areas (a%). Concentrated shrimp COs (1 g/L) significantly increased M% (average extension of fungal colonization in the root system) and A%—but not F%—and never significantly outperformed concentrated (nor diluted) fungal COs. Importantly, diluted shrimp COs (1 mg/L) achieved the overall lowest efficiency in AM promotion, with poor increases in F%, M%, m% (intensity of mycorrhization in colonized parts of the root) and A% and a significant increase compared to controls only for a%. Altogether, fungal COs promoted AM development more efficiently than shrimp COs, with a dose-dependent response.

## Discussion

The modifications we have introduced to the method by Di Mario et al.^24^, in order to reduce reagent use and processing time, provided a 61% increase in chitin recovery from *P. ostreatus* dry biomass, compared to the original article where the chitin recovery was 15.3% ± 2.2%. However, this value remains within the range described by Jones et al.^19^. Also chitin recovery from *C. bertholletiae* did not differ significantly from literature results^25^. Since literature data report a high chitosan/chitin ratio in mucoromycetes^26,27^, we were not surprised to extract a large amount of chitosan from *C. bertholletiae*, but—unexpectedly—this fungus also provided a relevant major amount of chitin, which was indeed about 46 times more abundant than chitosan in terms of percent yield. Lastly, a very high chitosan yield was also surprisingly obtained from *T. viride*, even if ascomycota cell walls are reported to be richer in chitin^26,27^. Such differences between expected and actual yields may be ascribed to several causes: growth conditions and strain-specific features may have a major role, but we cannot exclude that the changes we introduced to the extraction protocol differently affect chitin and chitosan extraction efficiency.

In addition to a considerable improvement in chitin extraction efficiency, our protocol also provided a very high yield in COs: a + 65% increase was achieved from acid hydrolysis of *C. bertholletiae* chitin, compared to the chitin acid hydrolysis from crab shell and squid pen performed by Kazami et al.^28^, while short chain CO recovery from *P. ostreatus* did not differ significantly from the expected yield^28^. The presence of different amounts of lipid, glucan or protein traces in the chitin powder may have affected CO hydrolysis and solubilisation efficiency, and account for such differences.

NMR analysis of extracted COs generated spectra with relatively broad and low peaks. Such features are often associated with polymers, whereas NMR spectra of oligomers generally have narrower and more defined spikes^29^. However, this consideration applies to purified solutions of homogeneous oligomers (e.g. tetramers); in our case, fungal chitin was randomly hydrolysed, producing a mix of COs of different length. This range of variability can explain the observed spectral features, as suggested by the analogous results of Kazami et al.^28^, who identified six peaks in GPC chromatography of water-extracted COs as N-acetyl-glucosamine monomers, di-chitooligosaccharides (CO_2_), tri-chitooligosaccharides (CO_3_), tetra-chitooligosaccharides (CO_4_), penta-chitooligosaccharides (CO_5_) and hexa-chitooligosaccharides (CO_6_) based on their retention times: the amounts of CO_4_, CO_5_ and CO_6_ recovered exceeded those of *N*-acetyl-glucosamine and CO_2_. The presence of CO_4_–CO_5_ oligomers in all our CO samples was also confirmed by HPLC–MS/MS analyses as described. However, considering that the samples were not subjected to further purification, we cannot completely exclude the presence of longer chain oligomers.

Beside glomeromycetes, other fungi have been reported to produce small amounts of LCOs^41^. Their presence in our extracts appears unlikely for several reasons: the relatively harsh treatments included in the extraction process are not supposed to preserve this type of molecules; furthermore, LCOs are poorly soluble in water and have only been detected using specific butanol extraction^41^. In this context, the small traces of acyl chains detected only in *T. viride* extracts by NMR analysis (peaks at 1.2 and 0.9 ppm in Supplementary Fig. S4 online) appears surprising and could be related to sample contamination (e.g. by paraffins used to grease the glassware junctions). Confirming the presence and origin of this anomalous signal goes beyond the scope of this work, also considering the low yield of *T. viride* (Table 1), which makes the use of this strain unpractical for low-cost production of chitinic molecules to be used for field-scale applications.

Among hydrolysed products, the highest acetylation degree (45%) was observed in *T. viride* COs. In addition, *T. viride* and *P. ostreatus* CO spectra showed narrower and clearer spikes than those of *C. bertholletiae* and shrimp COs, a possible clue of higher homogeneity in oligomer length in the former two samples. By contrast, shrimp-derived COs displayed the lowest acetylation degree (3.8%), which raised to 19.7% following our peracetylation treatment.

NMR analysis showed acetylation degree as the only major difference between fungal and shrimp COs. This is not surprising because chitin in arthropods and fungi presents the same α polymorphic form (the most abundant in nature), despite their very distant phylogenetic positions^18^; β-chitin occurring only in squid pen, sea tube worms, and some algae^30,31^. Significantly, the higher acetylation degree of fungal COs was associated with their higher activity as elicitors symbiotic signalling in the AM host plant. In line with this observation, our tests demonstrated that shrimp CO bioactivity was boosted upon peracetylation, with a significant enhancement of the nuclear Ca^2+^ spiking response (average number of peaks and percent of activated atrichoblasts). These converging results hint at acetylation as a fundamental feature for CO bioactivity in symbiotic signalling, shedding new light on the molecular basis of plant-fungus recognition in AM.

Plants have evolved refined receptorial mechanisms to perceive and discriminate between COs, with important differences between plant lineages. In Arabidopsis, long chain molecules (CO8) elicit defence responses when recognized by a homodimer of the plasma membrane-associated receptors-like kinase Chitin Elicitor Receptor Kinase 1 (*At*CERK1^32^); in rice, *Os*CERK1 has lost the ability to directly bind COs, but this function in defence elicitation is played by Chitin Elicitor Binding Protein (*Os*CEBiP^7^), which interacts with *Os*CERK1 in a hetero-tetrameric receptor complex^33,34^. Recently, Feng et al.^16^ showed the activation of defence and symbiotic signalling as a result of short chain COs perception by the *Mt*CERK1 and the *At*LYK5 (*A. thaliana* chitin receptor) homolog LYR4 in *M. truncatula*. They also observed that diverse combinations of stimuli and receptors, including the receptor-kinase *Mt*DMI2, might contribute to inhibit plant defence responses and to promote symbiosis signalling after CO perception^34^. A role in both symbiosis and immunity regulation has also been shown for the LysM receptor-like kinase *Mt*LYK9 by Gibelin-Viala et al.^35^. On the same line, a putative CO receptor with a strong affinity for short chain but not for longer chain COs has recently been characterized in rice as *Os*MYR1^36^. The *Os*MYR1/*Os*CERK1 heterodimer assembles and activates upon CO4 binding to *Os*MYR1, triggering symbiotic signalling through nuclear Ca^2+^ spiking^36^.

All such CO-binding proteins have extracellular domains containing three tandem LysM motifs (LysM1-3) that bind *N*-acetyl-glucosamine residues^37^. Detailed structural analyses revealed a crucial role for the second motif, LysM2, which discriminates chitin from other polysaccharides by interacting with *N*-acetyl groups^38^. Furthermore, the structure of chitin-bound *At*CERK1-ECD shows that saccharide units exhibit an alternation of 180° flipping along the chitin chain, with the acetyl groups from one side of the chitin chain being solvent-exposed, suggesting that full acetylation may not be necessary for maximal chitin interaction with *At*CERK1^39^, and possibly for all LysM domain receptors.

This scenario is fully consistent with our observation that all short chain COs tested were able to elicit symbiotic Ca^2+^ signals, but stronger bioactivity was recorded for COs with higher degrees of acetylation, in line with the key role of the N-acetyl group in receptor-ligand interaction. The relevance of acetylation degree of chitin, chitosan and their derivatives is also highlighted in other biological processes, such as the ability of wound recovery in animals, as described by Jones et al.^19^.

Chitin-derived molecules have previously been used in laboratory conditions, with beneficial effects on plant growth and stress tolerance^4^. As shown in the Results section, our CO treatments generally increase fungal colonization, in agreement with Volpe et al.^4^.

Our results provide a more detailed set of data on dose–response correlation regarding AM colonization on *M. truncatula*. Evidence shows that COs dose–response correlation is also demonstrated in the pre-symbiotic stage, as described by Oldroyd^13^, who compared Ca^2+^ spiking triggered by different molecules, including rhizobial Nod factors, LCOs (lipo-chitooligosaccharides) and short COs.

Rush et al.^41^ observed that COs slightly enhance spore germination in *Aspergillus fumigatus* and pseudohyphae formation in *Candida glabrata*, albeit to a significantly more limited extent, compared to LCOs^40^. Further analyses will be necessary to investigate if analogous effects are also present in AM fungi.

According to our results, the progressive enhancement of AM colonization in *M. truncatula* correlates with increasing concentrations of *P. ostreatus-*derived COs from 1 mg/L to 1 g/L. Further investigations will be needed to define the optimal concentrations for biologically relevant improvements in AM development for agricultural applications.

### Perspectives

Despite the positive results in protocol optimization, both extraction and hydrolysis protocols still appear to be considerably time-consuming and complex: further simplifications will have to be tested in the next experiments to shorten the total processing time, improve extraction yields and reduce costs. To this aim, the protocol could significantly be improved by reusing media and reagents, skipping a few redundant steps in the purification phase, combined with the possibility to use organic waste from industrial food processing activities as growth media.

Water-soluble chitin oligomers, in addition to their acknowledged role in stimulating the development of AM symbiosis^4^, have many additional applications, including lowering of blood cholesterol and blood pressure^41,42^, inducing protective effects against infections^43^ and enhancing antitumor properties and anti-adhesion activity^44–46^. They are also used in food and biomedical industries^47–49^. Furthermore, they are considered to be functional foods because of their non-digestibility by intestinal enzymes, which allows their use as prebiotics. They stimulate beneficial bacteria in the gastrointestinal tract (Bifidobacterium and Lactobacillus sp.)^50,51^. They can also act as thickeners and stabilizing agents^30^.

Consequently, large-scale and low-cost CO production is of great interest for industrial and agricultural application.

## Methods

### Fungi

The mucoromycete *Cunninghamella bertholletiae* MUT 2861, the basidiomycete *Pleurotus ostreatus* MUT 2976 and the ascomycete *Trichoderma viride* MUT 3170 were grown on malt extract broth (ME), containing 20 g/L glucose, 20 g/L malt extract, and 2 g/L peptone, as described by Spina et al.^52^. In more detail, fungi were inoculated as conidial suspension in 500 mL flasks containing 350 mL of medium and incubated at 24 ± 2 °C in the dark on an orbital shaker set at 130 rpm. After 12 days, the biomass was filtered, washed and lyophilized.

### Chitin purification

Chitin was purified from lyophilized biomass of *P. ostreatus*, *C. bertholletiae* and *T. viride*, using a modified version of the method described by Di Mario et al.^24^. Briefly, fungal biomass was deproteinated by treatment of 15 g of lyophilized mycelium with 500 mL of 1 N NaOH under vigorous stirring overnight at 40 °C. The suspension was centrifuged at 4500×*g* for 45 min and the supernatant, containing proteins and other impurities, was discarded. This treatment was repeated three times. The pellet, containing fungal wall polysaccharides, was suspended in 500 mL of boiling distilled water in a round-bottom flask equipped with a reflux condenser and stirred overnight at reflux in order to remove glucans. The suspension was then centrifuged at 4500×*g* for 45 min. The supernatant containing wall glucans was discarded. The pellet was washed three times with distilled water at 100 °C. In a round-bottom flask equipped with a reflux condenser, the pellet was treated with 500 mL of aqueous acetic acid (5%) and stirred for 3 h at 90 °C. After centrifugation at 4500×*g* for 45 min, the chitin pellet was separated from the supernatant (containing chitosan), washed three times with distilled water and lyophilized. Chitosan was precipitated by adding 1 N NaOH until pH reached 12 and centrifuged at 4500×*g* for 45 min. The resulting pellet was washed twice with distilled water and lyophilized. Fresh and dry weight of both extracted chitin and chitosan were measured before and after lyophilization.

### Chitooligosaccharide production

Chitin hydrolysis under acidic conditions was performed following a modified version of the method described by Kazami et al.^28^. Briefly, 1 g of lyophilized chitin for each fungus was hydrolysed in 30 mL of 37% HCl at 40 °C for 1 h. After cooling back to room temperature, the reaction mixture was then poured into 160 mL of acetone and stirred overnight at 4 °C. The solution was then centrifuged at 10,000×*g* for 10 min (4 °C) and the precipitate was washed with acetone until pH reached 5. Finally, the pellet was re-suspended in cold diethyl ether, centrifuged at 10,000×*g* for 10 min at 4 °C and dried under vacuum over P_2_O_5_.

The dried precipitate was soaked in 25 mL of distilled water and stirred overnight at 20 °C. Subsequently, the pellet was centrifuged at 15,000 g for 10 min (20 °C). Centrifugation produced a precipitate (which was soaked in 10 mL of distilled water and stirred overnight at 20 °C) and a supernatant (which was stored). The suspended precipitate was further centrifuged at 15,000 g for 10 min at 20 °C. The combined supernatant obtained for each fungus—expected to contain water-soluble chitin oligomers—was completely lyophilized to obtain a powder of purified chitooligomers.

### Shrimp CO peracetylation

In order to check for differences in the biological activity of COs with different acetylation degree, shrimp shell-derived COs (Zhengzhou Sigma Chemical Co., Ltd.—Zhengzhou, Henan, China) were N-acetylated following the protocol described by Trombotto et al.^53^.

100 mg of shrimp CO mixture were dissolved in 25 mL of methanol/water (50/50, v/v) solution and adding 50.3 mL of acetic anhydride. After stirring at room temperature for 5 min the solution was concentrated to dryness, the solid residue was re-dissolved in distilled water and evaporated under vacuum. This treatment was repeated twice, then the CO sample was dissolved in 50 mL of 0.01 M HCl and, after freeze-drying, isolated as a white powder.

### Chemical analyses

Extracted COs were initially analysed by DIMS on an LCQFleet Ion trap mass spectrometer (Thermo Fisher, USA) equipped with an electrospray source. Extracts were dissolved in MilliQ water (10 mg L^−1^) added with 20% of acetic acid:methanol (1:100, v/v) and filtered by 0.2 µm cellulose acetate syringe filter. The syringe pump was used at a flow rate of 10 mL min^−1^ to infuse samples directly into the mass spectrometer. The electrospray source was operated in positive ion mode, and the following source conditions were set: spray voltage at 3.5 kV, sheat gas flow at 20 arbitrary units, auxiliary and sweep gas flow at 0 arbitrary units, ion transfer temperature at 280 °C. The mass spectrometer was operated in full scan mode; exploring the scan range m/z 200–2000 and splitting the scan range in six scans (m/z 200–600, 500–900, 800–1200, 1100–1500, 1400–1800, 1700–2000).

COs were subsequently characterized by HPLC using an Ultimate 3000 (Dionex). Separation was performed using a hypercarb column (5 µm, 2 × 100 mm; Hypercarb, Thermo). Solutions of acetic acid:water (1/1000, v/v) and acetonitrile were pumped at 0.4 mL min^−1^. The gradient used was 100% acetic acid:water for 1 min, then 100% to 50% acetic acid:water in 30 min then 50% to 0% acetic acid:water in 3 min.

COs were identified in the multiple reaction monitoring (MRM) mode using a 4500 Q Trap mass spectrometer (Applied Biosystems, Foster City, CA, USA) with an electrospray ionization in the positive ion mode by monitoring the transitions from parent ion > common daughter ions (m/z: 204, 407, 610), and for quantification using the MRM 425 > 204 m/z (CO2), 628 > 204 m/z (CO3), 831 > 204 m/z (CO4), 1034 > 204 m/z (CO5). Mono-deacetylated (deAc) COs were identified in MRM mode by monitoring the transitions from parent ion > common daughter ions (m/z: 162, 365, 568), and for quantification using the MRM 383 > 162 m/z (deAc-CO_2_), 586 > 162 m/z (deAc-CO_3_), 789 > 162 m/z (deAc-CO_4_), 992 > 162 m/z (deAc-CO_5_). The capillary voltage was fixed at 5500 V, source temperature at 400 °C. Fragmentation was performed by collision-induced dissociation (CID) with nitrogen, collision energy 20 to 54 V, declustering potential 90 to 130 V depending on molecules^11^.

Furthermore, ^13^C CPMAS SSNMR^54–57^ and solution ^1^H NMR^58^, were used to obtain detailed structural information of both chitin and COs.

SSNMR spectra were acquired with a Bruker Avance II 400 Ultra Shield instrument, operating at 400.23 and 100.63 MHz, respectively for ^1^H and ^13^C nuclei. Samples were packed into cylindrical zirconia rotors with a 4 mm o.d. and an 80 µL volume. A small amount of sample (40 to 150 mg, depending on the sample) was collected from each batch and shredded into pieces, small enough to fill the rotor. ^13^C CPMAS spectra were acquired at a spinning rate of 12 kHz, using a ramp cross-polarization pulse sequence with a contact time of 3 ms, a 90° ^1^H pulse of 3.60 µs, optimized recycle delays between 1 and 2.1 s, for a number of scans between 400 and 3170, depending on the sample. For every spectrum, a two-pulse phase modulation (TPPM) decoupling scheme was used, with a radiofrequency field of 69.4 kHz. The ^13^C chemical shift scale was calibrated through the methylenic signal of external standard glycine (at 43.7 ppm).

Solution (D_2_O) ^1^H NMR spectra were acquired on a Jeol Eclipse 400 instrument, operating at 400.23 MHz for ^1^H nuclei. About 5 mg of each sample was dissolved in 600 µL of D_2_O and the solution was transferred in an NMR tube. The spectra were collected at room temperature. In order to ensure a complete relaxation of the magnetization, a relaxation delay of 60 s for 256 scans was employed for each spectrum.

### Bioactivity assay

The bioactivity of purified COs was analysed using an established test for the activation of the common symbiotic signalling pathway, based on live imaging of root epidermal cells in confocal microscopy^59^. Biological analyses were done using *Agrobacterium rhizogenes*-transformed ROCs of wild-type *Medicago truncatula* (genotype Jemalong A17) expressing the 35S:NupYC2.1 construct^60^. The ROC line, available in the lab^11^, was propagated in vertically-oriented Petri dishes containing M medium ^61^ and incubated at 25 °C in the dark for 10–14 days. Explants with consistent morphology and an identical number of lateral roots were chosen for all technical replicates.

Excised 1–2 cm-long lateral *M. truncatula* roots were treated with 1 mg/L solution of COs, extracted from each fungus (*P. ostreatus*, *C. bertholletiae* and *T. viride*); sterile distilled water was used as negative control and 1 mg/L solutions of short chain COs purified from shrimp shells (acetylated and non-acetylated) were used as positive control, based on previous studies^11,62^. A Leica TCS SP2 AOBS confocal laser-scanning microscope, equipped with a 40 × water-immersion objective, was used for live cell imaging of NupYC2.1 fluorescence in root atrichoblasts. Ratiometric analysis of NupYC2.1 FRET intensity over time was used to visualize variations in calcium concentration for each imaged nucleus during 30 min following the treatments. A minimum of 20 atrichoblasts from at least 6 independent root segments were analysed for each treatment.

### Pot cultures

The efficiency of *P. ostreatus* COs as stimulants of AM colonization was tested on pot-grown *M. truncatula* plants. Seeds were first scarified on sand paper in order to break the seed coat. They were sterilized using 5% sodium hypochlorite in water and washed several times in sterile distilled water. Seeds were then imbibed on 0.6% agar plates at 4 °C in the dark for 48 h to break dormancy, then moved at 23 °C for 5 days to allow germination. 10 days-old seedlings were transferred to 10 × 10 × 12 cm flowerpots with sterile quartz sand and grown for two weeks before further treatment.

1 g/L and 1 mg/L solutions were prepared in sterile distilled water using *P. ostreatus* COs (Po 1 g/L and Po 1 mg/L, respectively) or shrimp-derived COs (SH 1 g/L and SH 1 mg/L). Sterile distilled water was used for control treatments (CTR). All test solutions were added with 0.005% Tween 20 as a surfactant. Each plant was sprayed with 5 mL of test solution and the treatment was repeated 2 days later, prior to inoculation with the AM fungus *Funnelliformis mosseae,* (strain BEG 12) using a commercial inoculum (MycAgroLab, Bretenièr, France) mixed at 5% with sand.

Ten replicates were done for each of the following lines: CTR, Po 1 mg/L, Po 1 g/L, SH 1 mg/L and SH 1 g/L. During the first week, a plastic bag was placed on the flowerpots to prevent excessive desiccation and allow progressive plant acclimatisation. Plants were grown for 28 days in phytochamber under photoperiod of 16 h/day (23 °C) and 8 h/night (21 °C) and each plant was fertilized once a week with 20 mL of a modified Long Asthon nutrient solution containing 3.2 μM KH_2_PO_4_ as P source^63^.

Plants were harvested 28 days post inoculation and washed from sand before fresh weight of roots and shoots was determined. The shoots of 5 plants for each line were then dried at 60 °C until their weight was stabilised, to determine their dry weight (see Supplementary Fig. S5 online).

To determine the extension of fungal colonization, the remaining 5 plants for each test line were stained following the 'lactic blue' protocol developed by Trouvelot et al. (1986): root systems were excised, placed in 50 mL-tubes and submerged in the staining solution (0.1% cotton blue in lactic acid) for 12 h, then rinsed 2 times in water and 2 times in lactic acid (2 h for each rinsing) until all the excessive dye was removed. For microscope observation, each stained root system was cut randomly in 1 cm length pieces and distributed on 5 microscope slides (20 pieces for slide), until 1 m of root was collected for each plant.

The staining of intraradical fungal structures with lactic blue allowed both a morphological characterisation and a statistical quantification of root colonization according to the method by Trouvelot et al. (1986). Four quantitative parameters were calculated and used to compare the intensity of root colonization between samples. F%, colonization frequency in the root system, represents the percentage of segments containing intraradical fungal structures and is considered as a general indicator of the plant mycorrhizal status; M%, intensity of mycorrhizal colonization in the root system, reports on the average volume occupied by the fungus in each fragment, providing more detailed information on the extension of single infection units; m%, intensity of the mycorrhization in colonized parts of the root; a%, arbuscule abundance in colonized areas, indicates the average percentage of arbusculated cells within colonized segments and represents a reporter of symbiosis efficiency; A%, arbuscule abundance in the root system, estimates the average abundance of arbuscules in the whole root system, including non-colonized parts.

## Supplementary Information

Supplementary Information.

## Data Availability

The datasets generated during and/or analysed during the current study are available from the corresponding author on reasonable request.
