# Supplementary material for: Extraction of short chain chitooligosaccharides from fungal biomass and their use as promoters of arbuscular mycorrhizal symbiosis
Source: Sci Rep. 2021 Feb 15;11:3798. doi: 10.1038/s41598-021-83299-6 (PMC7884697; doi:10.1038/s41598-021-83299-6)
Supplement: Supplementary file 1 — Supplementary Information. [file 41598_2021_83299_MOESM1_ESM.pdf]

Supplementary Information

Extraction of short-chain chitooligosaccharides from fungal biomass and their use as promoters of arbuscular mycorrhizal symbiosis

Andrea Crosino[1], Elisa Moscato[1], Marco Blangetti[2], Gennaro Carotenuto[1], Federica Spina[1], Simone Bordignon[2], Virginie Puech-Pagès[3], Laura Anfossi[2], Veronica Volpe[1], Cristina Prandi[2], Roberto Gobetto[2], Giovanna Cristina Varese[1], Andrea Genre[1*]

[1] Department of Life Science and Systems Biology, University of Turin, Turin 10100, Italy

[2] Department of Chemistry, University of Turin, Turin 10100, Italy

[3] Laboratoire de Recherche en Sciences Végétales, Université de Toulouse, CNRS, UPS, Castanet-Tolosan 31320, France.

*Corresponding author at: Department of Life Science and Systems Biology, University of Turin, Turin 10100, Italy E-mail address: andrea.genre@unito.it (A. Genre).

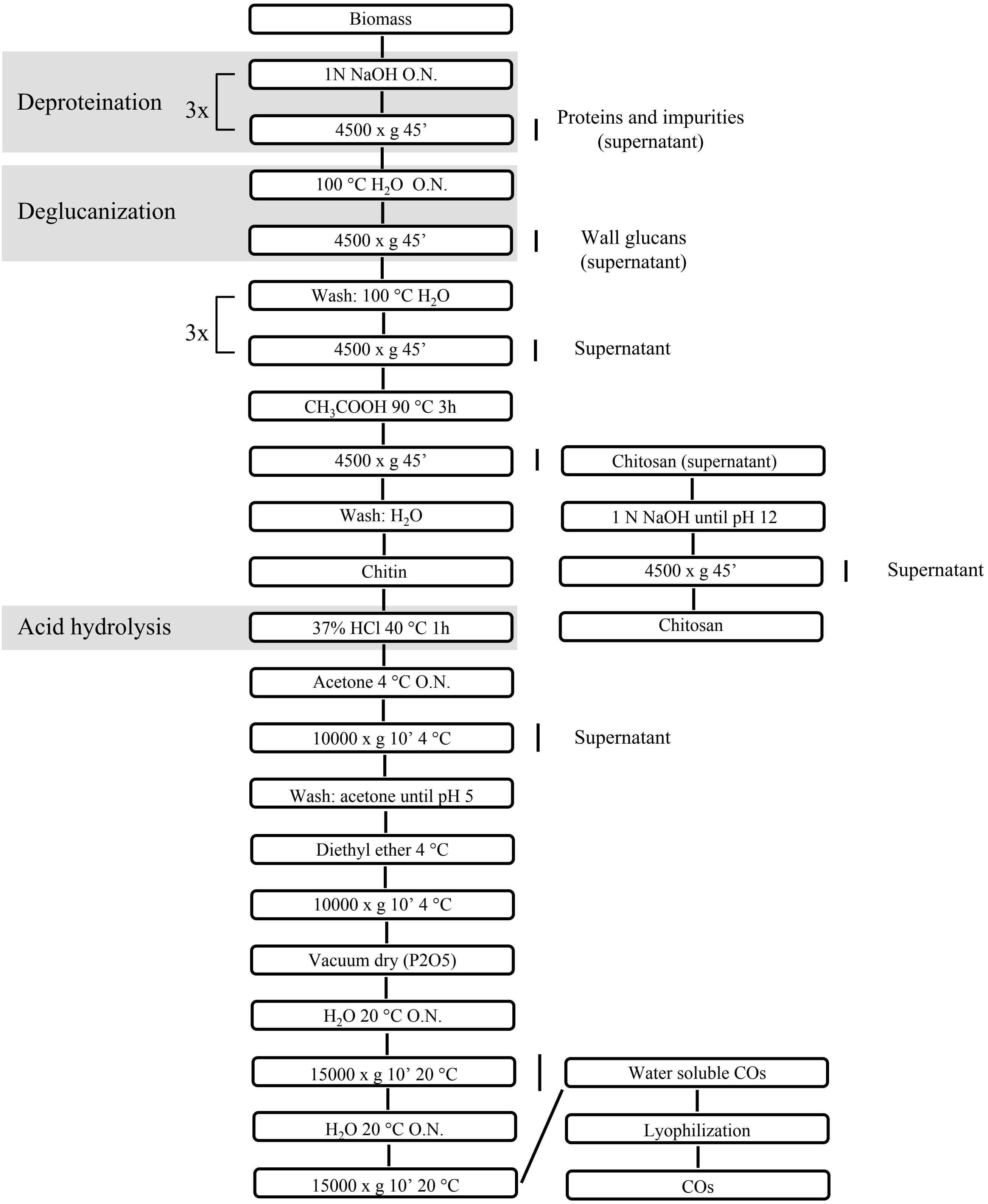

Supplementary Figure S1. Schematic representation of the CO extraction workflow. Lyophilized mycelial biomass was deproteinated, deglucanized prior to chitin separation from chitosan. COs were obtained by acid hydrolysis of chitin. H_2O in the figure refers to distilled water.

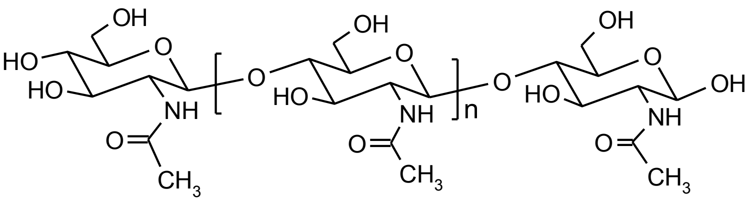

Fully acetylated short chain CO (n = 0 to 5)

	Fully acetylated	Mono-deacetylated	Di-deacetylated	Tri-deacetylated
P. ostreatus		383 (CO2)		
	628 (CO3)	586 (CO3)		
	831 (CO4)	789 (CO4)		
	1024 (CO5)	992 (CO5)		
	1227 (CO6)		1153 (CO6)	1111 (CO6)
C. berthollethiae	425 (CO2)	383 (CO2)		
	628 (CO3)	586 (CO3)	544 (CO3)	
	831 (CO4)	789 (CO4)	747 (CO4)	705 (CO4)
	1024 (CO5)	992 (CO5)	950 (CO5)	
	1227 (CO6)	1195 (CO6)	1153 (CO6)	1111 (CO6)
				1314 (CO7)
T. viride	425 (CO2)	383 (CO2)		
	628 (CO3)	586 (CO3)		
	831 (CO4)	789 (CO4)		
	1024 (CO5)	992 (CO5)		
	1227 (CO6)	1195 (CO6)	1153 (CO6)	
	1430 (CO7)	1398 (CO7)		

Supplementary Figure S2. Pseudomolecular ions of COs identified by DIMS in positive mode. The M+H values for the CO (and corresponding CO_n) identified in each sample extract are listed. Fully acetylated and mono-deacetylated COs (n=2 to 7) were shown for almost all extracts. In addition, several di- and tri-deacetylated COs (n=3 to 7) were present in the extract obtained from *C. berthollethiae*

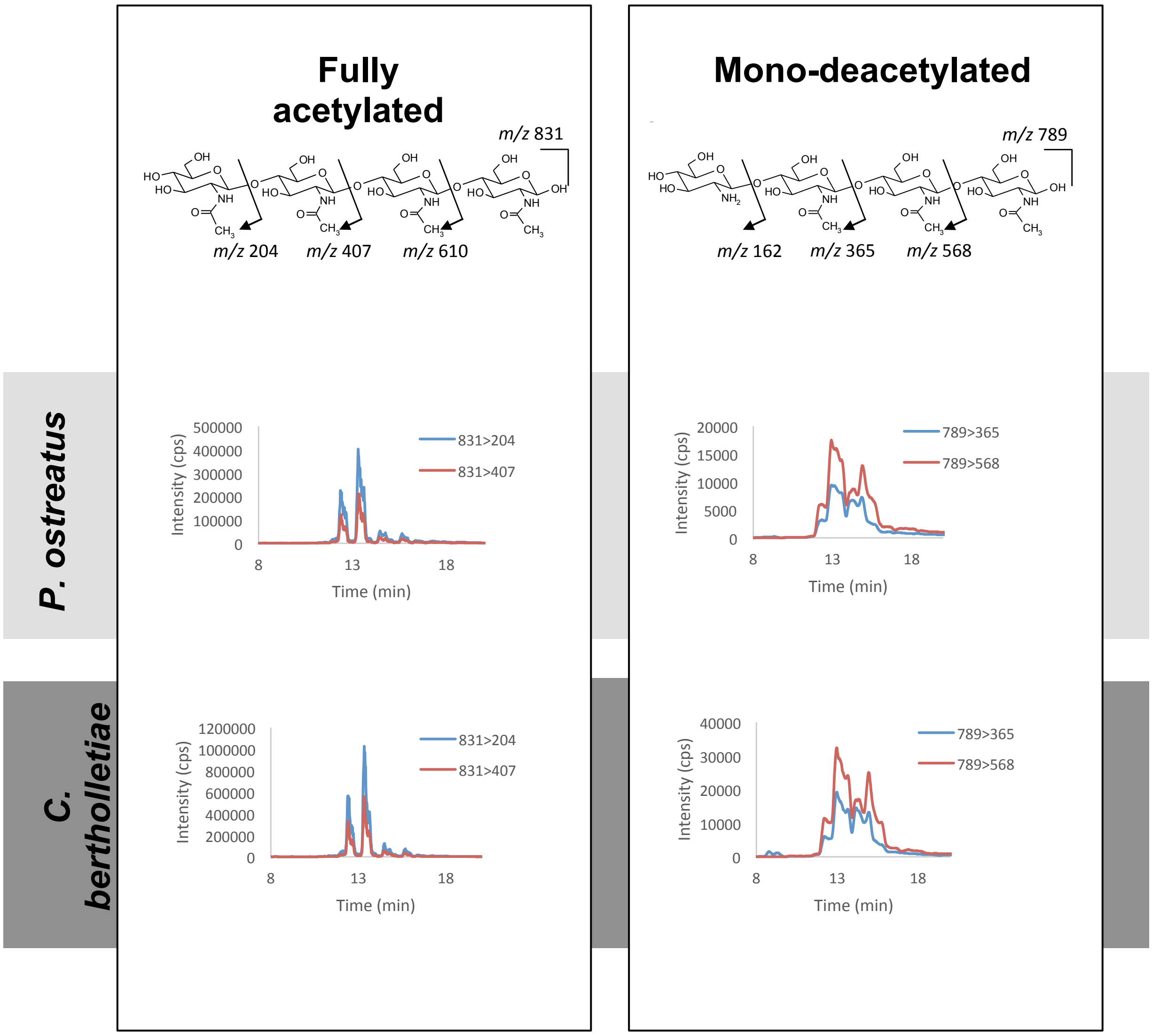

Supplementary Figure S3. HPLC-MS/MS identified fully acetylated and mono-deacetylated COs in *P. ostreatus* and *C. bertollethiae* extraction products. Schemes in the top row present the structure of fully acetylated (left) and mono-deacetylated CO4 (right), including calculated precursor and product B ions in positive mode (M+H)**.**

HPLC-MS/MS chromatograms in MRM mode are presented for *P. ostreatus* and *C. bertholletiae* CO samples. Two transitions (shown in red and blue, respectively) are presented for each sample. In all cases, two peaks are detected corresponding to the molecule alpha and beta anomers. In more detail, peaks were detected at 12.5 and 13.5 min for fully acetylatd COs and at 13 and 14,5 min for mono-deacetylated COs.

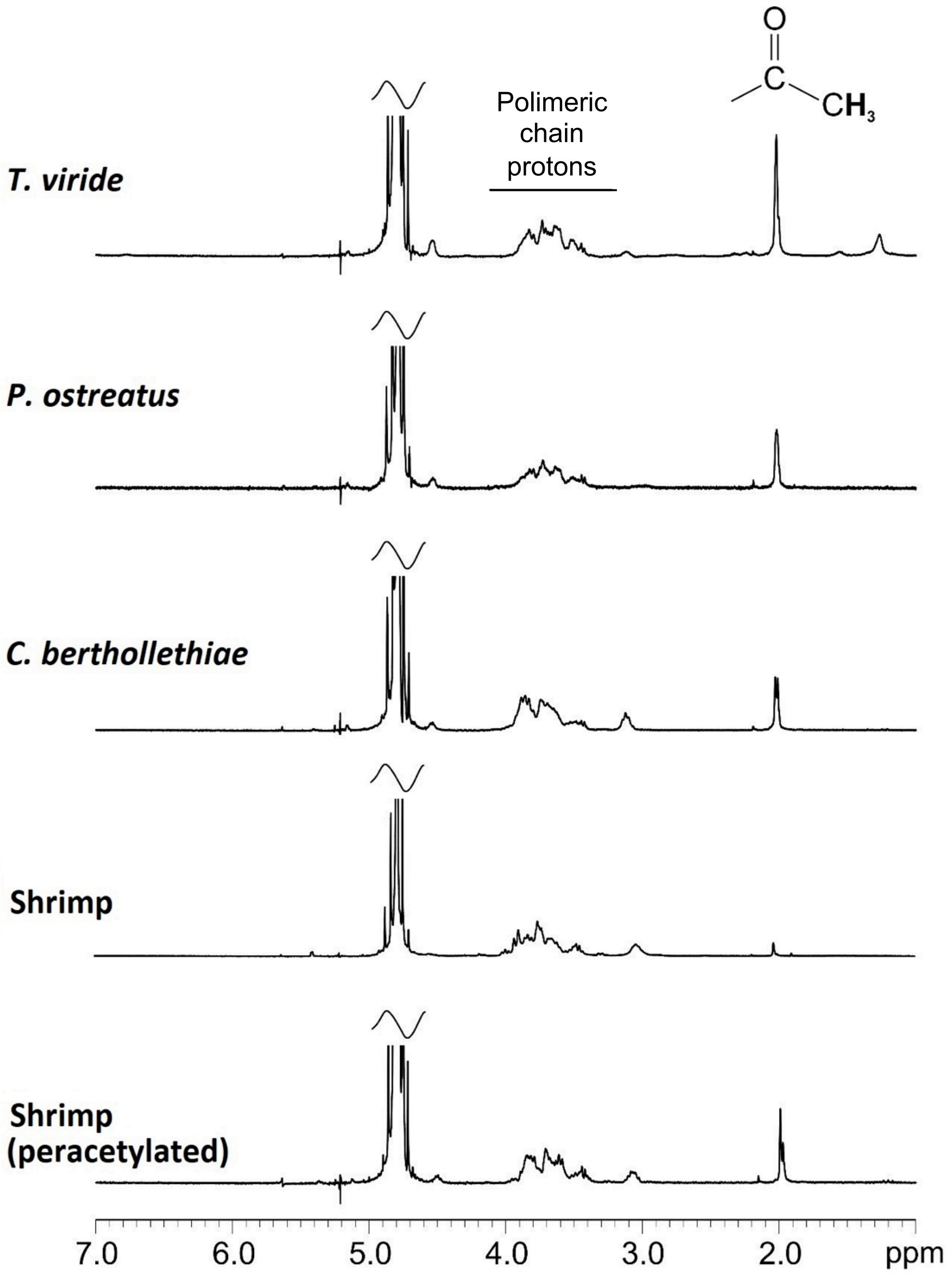

Supplementary Figure S4. ^{1}H solution (D_2O) NMR spectra of COs from *T. viride*, *P. ostreatus*, *C. berthollethiae* and shrimps (both untreated and peracetylated). The signal at ~4.7 ppm can be ascribed to the solvent and is represented as out-of-scale.

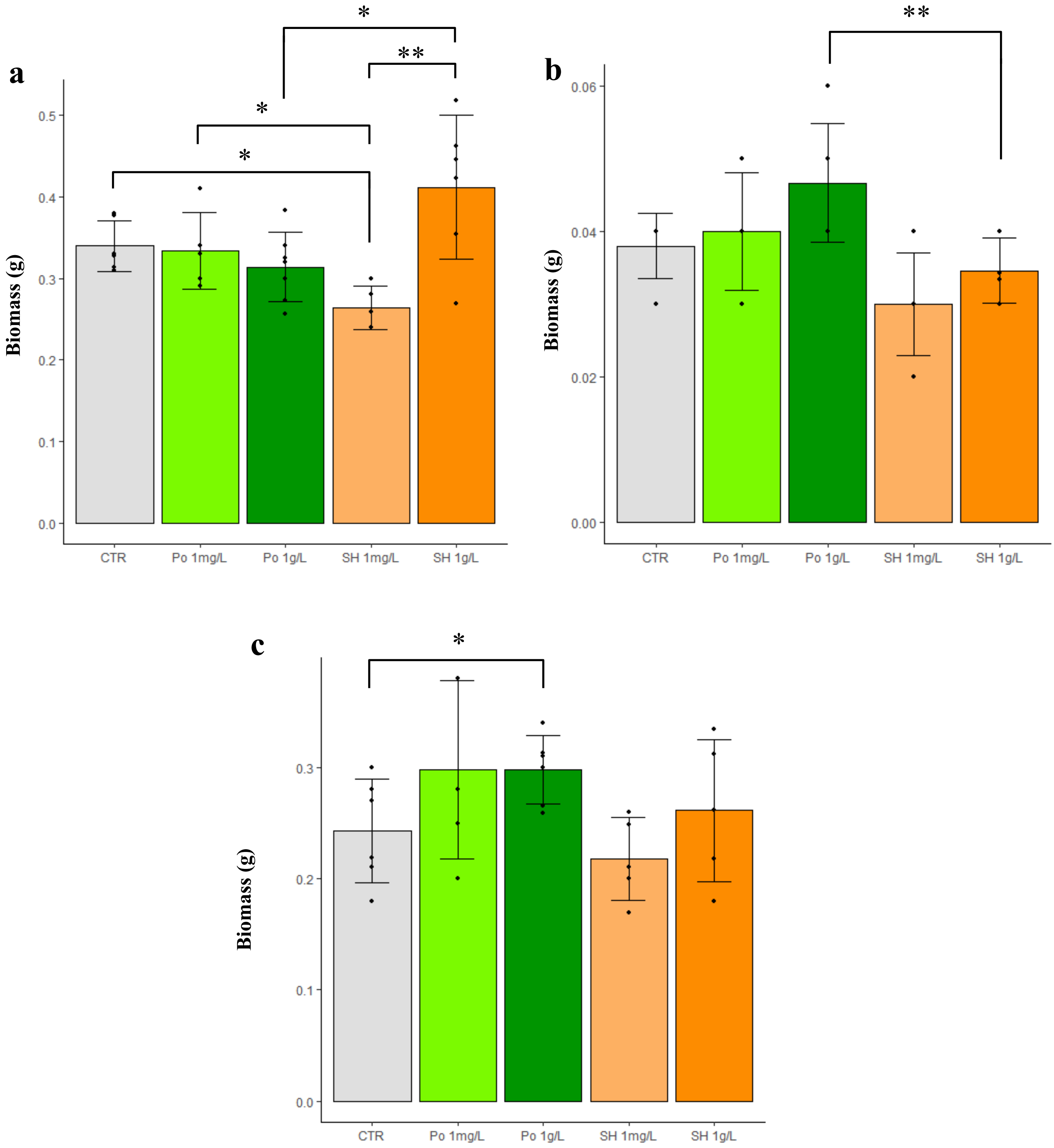

Supplementary Figure S5. Root wet biomass **a**, shoot dry **b** and wet **c** biomass of mycorrhizal *M. truncatula* plants treated with different CO solutions. A significant increase in shoot biomass, compared to control, was only observed after the application of 1 g/L solution of *P.ostreatus* COs. Despite the greatest amount of root wet biomass provided by the treatment with 1 g/L crustacean COs, no significant differences were observed compared to control plants. CTR= water-treated control; Po 1mg/L= *P. ostreatus* COs 1 mg/L; Po 1g/L= *P. ostreatus* COs 1 g/L; SH 1mg/L= crustacean COs 1 mg/L; SH 1g/L= crustacean COs 1 g/L. A minimum of four biological replicates were evaluated for each treatment. Student's t test: *P < 0.05, **P < 0.01.
